# Causal association between Parkinson disease, gut microbiota, and constipation: A Mendelian study

**DOI:** 10.1097/MD.0000000000045552

**Published:** 2025-10-31

**Authors:** Jiamu Zheng, Beibei Xu, Lidan Gao, Wenhuan Wang

**Affiliations:** a The Second Clinical Medical College, Zhejiang Chinese Medical University, Hangzhou, Zhejiang, China; b Department of Gastroenterology, Wenzhou Third Clinical Institute Affiliated to Wenzhou Medical University, Wenzhou People’s Hospital, Wenzhou, Zhejiang, China; c Department of Research Laboratory Center, Wenzhou Third Clinical Institute Affiliated to Wenzhou Medical University, Wenzhou People’s Hospital, Wenzhou, Zhejiang, China.

**Keywords:** constipation, gut microbiota, GWAS, Mendelian randomization, Parkinson disease

## Abstract

This study utilized multiple 2-sample Mendelian randomization (MR) approaches to evaluate the causal effects of gut microbiota on the risk of constipation and Parkinson disease (PD) and further investigated the mediating role of gut microbiota in the pathway linking PD to constipation. Two-sample MR analysis was conducted to identify gut microbiota with significant causal associations with constipation. These identified gut microbiota, along with PD, were incorporated as exposure variables into a MVMR framework. A comprehensive model encompassing gut microbiota, PD, and constipation was then established to perform mediation analysis, aiming to quantify the indirect effect of PD on constipation through gut microbiota. The 2-sample MR analysis identified statistically significant causal associations (*P* <.05) between specific gut microbiota and the development of constipation. Notable findings included *c_Betaproteobacteria* (OR = 0.899188), *c_Methanobacteria* (OR = 0.950024), *f_Methanobacteriaceae* (OR = 0.950024), *g_Eubacterium rectale group* (OR = 0.893372), and *o_Methanobacteriales* (OR = 0.950024). In the MVMR analysis, the association between PD and constipation was significant in Models 1 and 5 (*P* <.05). Mediation effect analysis revealed that the *Eubacterium rectale group (g_Eubacterium rectale group*) and *Methanobacteriales (o_Methanobacteriales*) exerted an indirect influence on the development of constipation through PD, with mediation effect proportions of 193.17% and 128.44%. This study revealed the potential causal effects of specific gut microbiota on constipation and, for the first time, proposed a mediating mechanism whereby PD indirectly influences constipation through certain gut microbiota. These findings provide novel insights into the complex relationships among PD, gut microbiota, and constipation, offering potential targets for gut microbiota-based interventions to address PD-associated constipation.

## 1. Introduction

Parkinson disease (PD) is a common neurodegenerative disorder of the central nervous system. However, its clinical manifestations extend beyond motor impairments, encompassing a wide spectrum of non-motor symptoms that substantially affect patients’ quality of life. Among these, gastrointestinal dysfunction is particularly prominent, with constipation representing one of the most frequent and burdensome non-motor features.^[1]^ Constipation, in general, is a prevalent gastrointestinal symptom characterized by reduced bowel movements, decreased stool volume, hard stools, and difficulty in defecation.^[2,3]^ The consequences of constipation include anal fissures, hemorrhoids, an increased risk of colorectal cancer, cardiovascular events, and even sudden death.^[4–6]^ The pathogenesis of constipation is multifactorial and involves dysbiosis of the gut microbiota, emotional disturbances, and autonomic dysfunction.^[7–9]^ Thus, constipation constitutes an important clinical problem both in the general population and in patients with chronic neurological conditions.

In the context of PD, constipation acquires additional clinical and mechanistic relevance. Epidemiological studies estimate that more than 20% of PD patients suffer from constipation, which may precede motor manifestations by years and is recognized as an early prodromal marker.^[10,11]^ Neuropathological investigations have revealed early α-synuclein(α-Syn) deposition in the enteric nervous system, vagus nerve, and dorsal motor nucleus, contributing to impaired autonomic regulation of gut motility.^[12]^ Degeneration of parasympathetic pathways and myenteric plexus abnormalities further delay intestinal transit, while gut microbiota dysbiosis alters mucosal immunity, barrier function, and neurotransmitter metabolism, collectively exacerbating bowel dysfunction.^[13,14]^ Importantly, constipation severity in PD patients has been positively correlated with the severity of motor symptoms, depressive symptoms, and the dosage of anti-PD medications, underscoring its potential role as both a marker and a modifier of disease progression.^[8,15]^

In recent years, research on constipation has shifted from exploring isolated etiologies to a more comprehensive evaluation of interactions among multiple factors. Notably, the abnormal effects of PD on the structure and function of gut microbiota may influence host metabolism and neurological functions through the gut-brain axis, potentially contributing to the development of constipation. Furthermore, the onset and exacerbation of constipation have been found to correlate with the progression of PD.^[16]^ Constipation itself may also lead to changes in gut microbiota composition, which could in turn affect the gut-brain axis and contribute to the pathogenesis of PD.^[17]^ Despite these insights, studies exploring the complex interactions between PD, gut microbiota, and constipation remain limited. Most existing research focuses on isolated mechanisms, lacking a systematic evaluation of the causal relationships among these 3 factors.

To address these gaps, causal inference methods such as Mendelian randomization (MR) and mediation analysis have gained traction in gastrointestinal and neurological research.^[18]^ MR analysis leverages the natural random allocation of genetic variants, mimicking the design of randomized controlled trials and offering a means to address confounding factors commonly encountered in observational studies.^[19]^ By integrating mediation analysis, researchers can delve deeper into the mechanistic interplay between PD-induced changes in gut microbiota and the onset and progression of constipation. This approach allows for the identification of potential biological processes and pathogenic pathways underlying these interactions, providing valuable insights into their complex relationships.

This study aims to leverage genome-wide association study (GWAS) data and utilize genetic variants as instrumental variables (IV) to evaluate the causal relationships between gut microbiota, PD, and constipation through an integrative approach combining MR and mediation analysis. Specifically, the study seeks to assess the impact of PD on gut microbiota and its subsequent influence on the risk of constipation, aiming to determine whether these relationships are causal. Currently, the clinical characteristics and underlying mechanisms of constipation in PD patients remain poorly understood, necessitating further research into its pathophysiology. This study explores whether constipation symptoms in PD patients are associated with gut microbiota dysbiosis, with the goal of providing novel insights into the pathogenesis of PD-related constipation. Such insights could inform future therapeutic and preventive strategies. By harnessing the complementary strengths of MR and mediation analysis, this research seeks to uncover a more precise causal relationship network underlying constipation, ultimately offering personalized and evidence-based guidance for the management of PD-associated constipation.

## 2. Materials and methods

### 2.1. Study guidelines and research design

This study employed a 2-sample MR approach, utilizing publicly available datasets to assess the impact of gut microbiota on the risk of developing constipation and to explore the potential mediating role of gut microbiota in constipation associated with PD. The research report adheres to the guidelines outlined in the “Strengthening the Reporting of Observational Studies in Epidemiology Using MR” (STROBE-MR) statement.^[20]^

**Figure 1.**
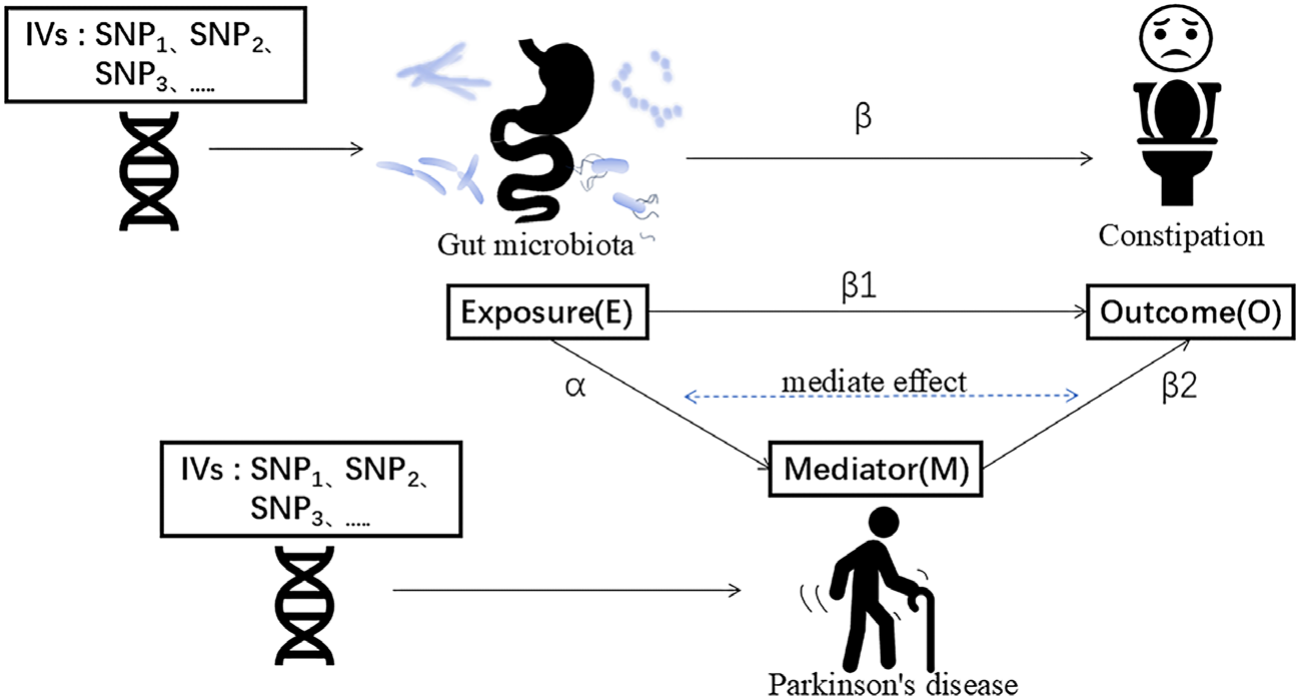
Technical schematic: the application of MVMR to investigate the causal relationship between PD, gut microbiota, and constipation. (β: the total effect of the exposure on the outcome. α: the regression coefficient of the exposure on the mediator. β2: the regression coefficient of the mediator on the outcome. The mediation effect is α*β2. β1: the direct effect which derived from the total effect minus the mediation effect. The mediation effect percentage is (α*β2/ β)*100%). PD = Parkinson disease.

The application of MVMR to investigate the causal relationship between PD, Gut Microbiota, and Constipation. The method integrates genetic variants associated with Gut Microbiota (exposure variable), evaluates their influence on PD (mediating factor), and assesses subsequent effects on Constipation (Fig. 1).

### 2.2. Data source

The Gut Microbiota GWAS data, which includes datasets for screening IV and exposures as well as comprehensive GWAS summary statistics for investigating the correlation between host genes and gut microbiota composition, were obtained from the GWAS Catalog website (https://www.ebi.ac.uk/gwas).^[21]^ Under the leadership of Kurilshikov and the MiBioGen consortium, genome-wide genotyping and 16S fecal microbiome data from 18,340 individuals across 24 cohorts were curated and analyzed. The analysis revealed substantial variability in microbial composition across cohorts: out of 410 genera, only 9 were detected in more than 95% of the samples. A GWAS focusing on host genetic variation in microbial taxa identified 31 loci that significantly influence the microbiome, reaching a genome-wide significance threshold (*P* <5 × 10^−8^).

The diagnosis of constipation is mainly based on clinical diagnostic criteria and related medical evaluation. The most commonly used criteria is the 10th edition of the International Classification of Diseases (ICD-10). According to the diagnostic criteria of ICD-10: K59.0, the diagnosis of constipation is mainly a state of hard and dry stool or difficult defecation with reduced frequency of defecation. For constipation GWAS data, the analysis was based on Round 9 results from the FinnGen Consortium, comprising 44,590 cases and 409,143 controls.

The diagnosis of PD is primarily based on clinical diagnostic criteria and relevant medical evaluations, with the most widely used standard being the International Classification of Diseases, 10th Revision (ICD-10). According to ICD-10: G20 diagnostic criteria, the hallmark of PD is bradykinesia, accompanied by at least 1 additional motor symptom such as rigidity, resting tremor, or postural instability. Additionally, there are non-motor symptoms that support the diagnosis but are not definitive, such as cognitive decline or dementia in later stages, depression, anxiety, sleep disturbances, and autonomic dysfunctions (e.g., constipation and urinary difficulties). For PD GWAS data, relevant PD traits were extracted from the Round 9 GWAS analysis of the FinnGen dataset using the keyword “Parkinson” to identify PD-related characteristics in the Finnish population. After excluding duplicate and confounding traits, 2 PD-related indicators (“Parkinson” and “PD strict definition”) were retained. These indicators were standardized, and further analyses were conducted using the R package “TwoSampleMR.”

### 2.3. Criteria for selection of instrumental variables

In this study, the criteria for selecting single nucleotide polymorphisms (SNPs) as IV were as follows: SNPs with a *P*-value less than 1 × 10^−5^ in the exposure GWAS were selected as potential instruments. SNPs exhibiting linkage disequilibrium (*r*² <0.001) and separated by a physical distance of more than 10,000 kb between any 2 genes were excluded. Subsequently, outcome GWAS data were extracted based on the selected SNPs. The *F*-statistic was also calculated to assess the strength of the instruments. An *F*-value <10 indicates that the genetic variants are weak instruments, which could introduce bias into the results.^[22]^ Therefore, SNPs with an *F*-value below 10 were excluded to minimize potential bias in the study’s conclusions.

### 2.4. Estimation of causal effects using Mendelian randomization

Several 2-sample MR methods were employed to evaluate the causal effects of gut microbiota on the risk of developing constipation. These methods included the inverse-variance weighted (IVW) method, MR-Egger method, weighted median method, simple mode, and weighted mode. In the absence of pleiotropy, the IVW method was utilized as the primary analytical approach, with the other methods serving as supplementary analyses. When heterogeneity was present, the IVW random effects model was applied. In cases where pleiotropy was detected, the MR-Egger method was used to estimate the causal effects. Additionally, the Steiger directionality test, provided by the TwoSampleMR package, was employed to assess the direction of causality.

### 2.5. Sensitivity analysis

Cochran *Q* test was utilized to evaluate heterogeneity among the SNP estimates. A statistically significant result from Cochran *Q* test suggests substantial heterogeneity in the analysis outcomes.^[23]^ For analyses showing significant heterogeneity, a random effects model for IV was employed to estimate the causal effect. The MR-Egger method was applied to assess the presence of pleiotropy in the IV. A *P*-value <.05 for the MR-Egger intercept indicates significant horizontal pleiotropy among the genetic variants.^[24]^ To assess the influence of each SNP on the relationship between constipation and gut microbiota, a leave-one-out sensitivity analysis was performed. A significant deviation in the MR effect estimate when excluding a specific SNP suggests that the MR effect estimate is sensitive to that particular SNP.

### 2.6. Multivariable MR analysis and mediation effect estimation

MVMR extends the traditional MR framework by incorporating genetic variants associated with multiple potentially interrelated exposures. This approach enables the estimation of the effects of multiple exposures on a single outcome, allowing for the direct evaluation of each exposure’s specific impact on the outcome. Prior to conducting MVMR analyses, univariable MR analyses were performed on gut microbiota and PD indicators that demonstrated a significant causal effect on constipation. Gut microbiota and PD factors showing significant causal relationships were subsequently selected as exposure variables. Separate MVMR models were then constructed for each gut microbiota and PD factor to evaluate their respective relationships with constipation. This methodology allows for the estimation of the direct effects of gut microbiota and PD on constipation, as well as the indirect effects of gut microbiota on constipation mediated through PD. These indirect effects were derived by combining the direct effect estimates of gut microbiota on PD indicators from univariable MR analysis with the direct effect estimates of PD indicators on constipation development from MVMR analysis. Given the complexity of mediation effects, this study focuses on discussing mediation only when there is a significant causal relationship between the exposure and outcome variables, as well as between the exposure and the mediator.

### 2.7. Statistical analysis

All data computations and statistical analyses were performed using R programming (version 4.4.1) [https://www.r-project.org/]. MR analyses were conducted using the TwoSampleMR package, and the robustness and reliability of the results were assessed through Cochran *Q* test and leave-one-out sensitivity analysis.^[25]^ Genetic pleiotropy was evaluated using the MR-Egger intercept method, while causal directionality was determined using the Steiger directionality test provided by the TwoSampleMR package. In the MR analysis of exposures related to constipation, odds ratios with 95% confidence intervals (95% CI) were used as evaluation metrics. All statistical tests were 2-tailed, and significance was set at *P* <.05.

## 3. Results

### 3.1. Instrumental variable selection

Based on the criteria for selecting IV in this study, SNPs with linkage disequilibrium or those inconsistent with the constipation GWAS data were excluded, while SNPs associated with gut microbiota were included. The selection results of IV for each measure are presented in Table 1, which lists only the key variables used in the MR analysis. All the F-statistics for these IV exceeded 10, indicating that the SNPs used in this study mainly serve as stable instruments with large effect sizes, thereby minimizing potential bias introduced by weak instruments.

**Table 1:** Instrumental variable screening and instrumental variable strength F test for gut microbiota and constipation.

Exposure	Number of SNPs	Median of *F*	Minimum of *F*	Maximum of *F*
*c_Betaproteobacteria*	10	21.562152	18.600512	24.775447
*c_Methanobacteria*	9	21.717305	19.856194	26.761492
*f_Methanobacteriaceae*	9	21.717305	19.856194	26.761492
*g_Coprococcus2*	8	20.273630	19.273037	23.994774
*g_Eubacterium brachy group*	10	20.612922	19.844893	22.177117
*g_Eubacterium rectale group*	8	20.792596	19.348749	26.652423
*g_Haemophilus*	9	21.814696	21.436680	29.341900
*g_Holdemanella*	11	21.426285	19.850406	24.517587
*g_Ruminococcus gnavus group*	11	20.933916	19.297110	29.660796
*unknown genus*	11	20.458591	18.650176	23.563578
*o_Methanobacteriales*	9	21.717305	19.856194	26.761492

SNP = single nucleotide polymorphism.

### 3.2. Mendelian randomization causal effect estimation

**Figure 2.**
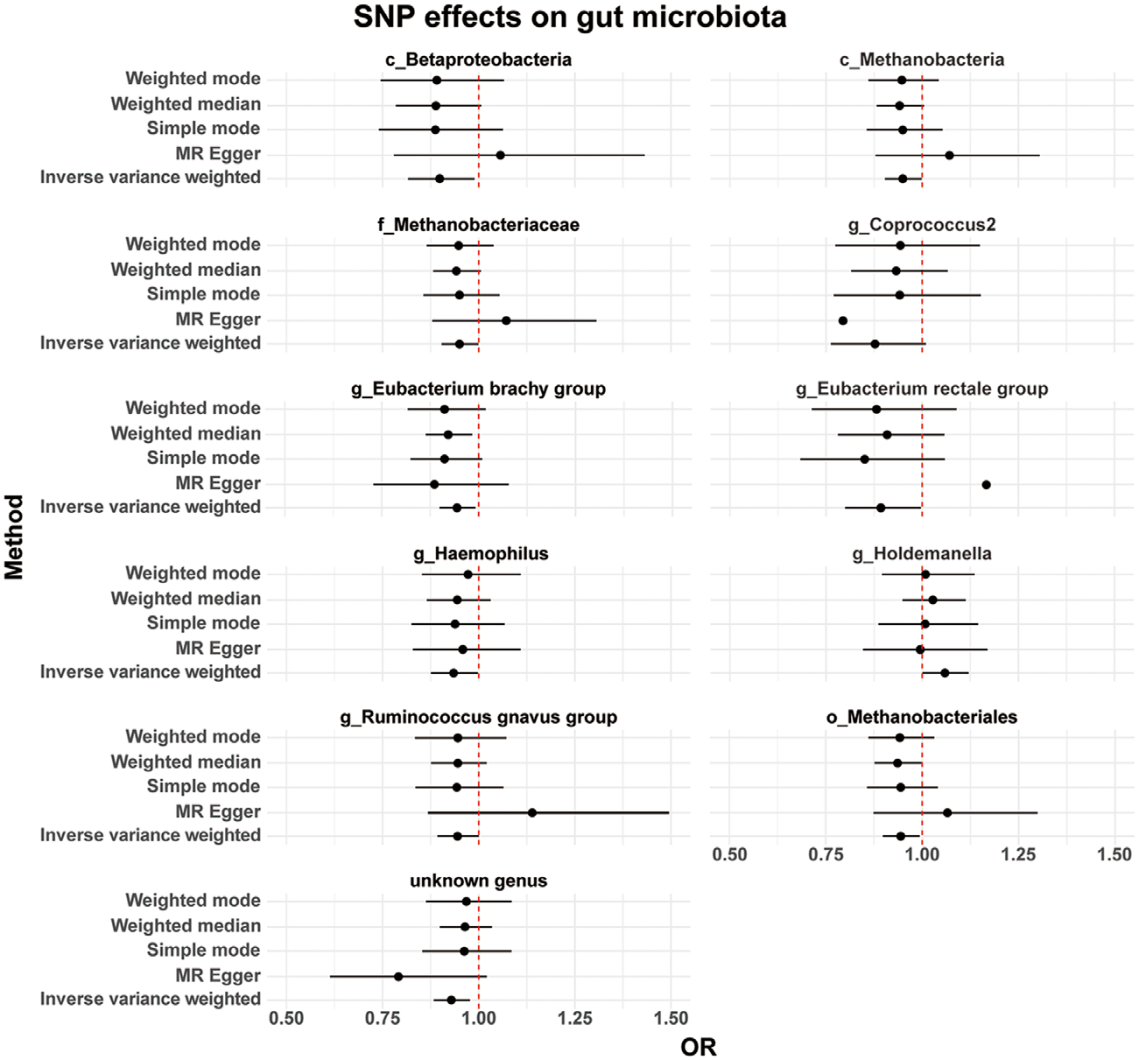
Effect estimates of different models in the Mendelian randomization analysis of gut microbiota on constipation. Causal effect estimates (OR, with 95% CI) are presented for 11 gut microbiota. All gut microbiota shown were initially selected based on statistical significance (*P* <.05) using the IVW method in single-exposure MR analyses. For each selected taxon, additional MR approaches (MR-Egger, weighted median, weighted mode, and simple mode) are also displayed for comparison. Points indicate causal estimates, and horizontal lines represent 95% confidence intervals. The vertical dashed line corresponds to the null value (OR = 1). Particular attention was given to 5 microbiota highlighted in the subsequent MVMR analysis – *g_Eubacterium rectale group, c_Betaproteobacteria, c_Methanobacteria, f_Methanobacteriaceae*, and *o_Methanobacteriales* – which were prioritized for further investigation of their potential causal role in constipation. CI = confidence interval, IVW = inverse-variance-weighted, MR = Mendelian randomization, MR-Egger = Mendelian randomization-Egger, MVMR = multivariable Mendelian randomization, OR = odds ratio.

The analysis was conducted using 5 models: MR-Egger, Weighted Median, IVW, Simple Mode, and Weighted Mode. Significant causal effects were identified using the IVW model (*P* <.05). The estimated causal effects for all 5 models are presented in Table 1. A forest plot of the effect estimates for the selected SNPs is shown in Figure 2, demonstrating that the distribution of fitted points is generally consistent across the 5 models. Although methods like MR-Egger can address bias in IV, they typically have lower statistical efficiency compared to the IVW model. Therefore, in the absence of substantial bias, the IVW method was used as the primary reference in this study.

Using the selected IVW model, the study revealed significant causal relationships between the risk of developing constipation and specific gut microbiota (*P* <.05) (Table 2). These gut microbiota include *c_Betaproteobacteria* (OR = 0.899188, 95% CI: 0.817092–0.989530, *P* = .029595), *c_Methanobacteria* (OR = 0.950024, 95% CI: 0.903459–0.998989, *P* = .045562), *f_Methanobacteriaceae* (OR = 0.950024, 95% CI: 0.903459–0.998989, *P* = .045562), *g_Coprococcus2* (OR = 0.862093, 95% CI: 0.747331–0.994478, *P* = .041754), *g_Eubacterium brachy group* (OR = 0.944298, 95% CI: 0.899416–0.991419, *P* = .021063), *g_Eubacterium rectale group* (OR = 0.893372, 95% CI: 0.800413–0.997123, *P* = .044293), *g_Haemophilus* (OR = 0.934609, 95% CI: 0.875588–0.997609, *P* = .042162), *g_Holdemanella* (OR = 1.059889, 95% CI: 1.001740–1.121414, *P* = .043344), *g_Ruminococcus gnavus group* (OR = 0.944809, 95% CI: 0.892720–0.999937, *P* = .049743), *unknown genus* (OR = 0.928714, 95% CI: 0.882869–0.976939, *P* = .004193), and *o_Methanobacteriales* (OR = 0.950024, 95% CI: 0.903459–0.998989, *P* = .045562). The inclusion of both confidence intervals and p-values allows for a more precise interpretation of the magnitude and statistical robustness of these associations.

**Table 2:** Mendelian randomization causal effect estimates of gut microbiota and constipation (using the IVW random effects model).

Exposure	Outcome	Number of SNPs	β	Standard error	*P*-value	OR 95 CI
*c_Betaproteobacteria*	Constipation	10	−0.106264	0.048846	.029595	0.899187 (0.817092–0.989530)
*c_Methanobacteria*	Constipation	9	−0.051268	0.025641	.045562	0.950024 (0.903459–0.998989)
*f_Methanobacteriaceae*	Constipation	9	−0.051268	0.025641	.045562	0.950024 (0.903459–0.998989)
*g_Coprococcus2*	Constipation	8	−0.148392	0.072885	.041754	0.862093 (0.747331–0.994478)
*g_Eubacterium brachy group*	Constipation	10	−0.057314	0.024845	.021063	0.944298 (0.899416–0.991419)
*g_Eubacterium rectale group*	Constipation	8	−0.112752	0.056059	.044293	0.893372 (0.800413–0.997123)
*g_Haemophilus*	Constipation	9	−0.067627	0.033282	.042162	0.934609 (0.875588–0.997609)
*g_Holdemanella*	Constipation	11	0.058165	0.028789	.043344	1.059889 (1.001740–1.121414)
*g_Ruminococcus gnavus group*	Constipation	11	−0.056772	0.028933	.049743	0.944809 (0.892720–0.999937)
*unknown genus*	Constipation	11	−0.073955	0.025829	.004193	0.928714 (0.882869–0.976939)
*o_Methanobacteriales*	Constipation	9	−0.051268	0.025641	.045562	0.950024 (0.903459–0.998989)

CI = confidence interval, IVW = inverse-variance-weighted, OR = odds ratio, SNP = single nucleotide polymorphism.

Finally, the Steiger directionality test was employed to validate the accuracy of the causal direction between gut microbiota and constipation (Table 3). The Steiger test calculated the variance explained (r²) by SNPs for both exposure and outcome, showing that the variance explained by SNPs for exposure was greater than that for the outcome. All directions were found to be TRUE, with *P*-values <.05, indicating that the causal directions were significantly accurate.

**Table 3:** Steiger directionality test for Mendelian randomization analysis of the effect of gut microbiota on constipation.

Exposure	Outcome	SNP *r*^2^ exposure	SNPs *r*^2^ outcome	Correct causal direction	Steiger *P*-value
*c_Betaproteobacteria*	Constipation	0.015314	1.70 × 10^−05^	TRUE	1.57 × 10^−45^
*c_Methanobacteria*	Constipation	0.013635	2.04 × 10^−05^	TRUE	2.99 × 10^−40^
*f_Methanobacteriaceae*	Constipation	0.013635	2.04 × 10^−05^	TRUE	2.99 × 10^−40^
*g_Coprococcus2*	Constipation	0.011667	5.92 × 10^−05^	TRUE	1.85 × 10^−32^
*g_Eubacterium brachy group*	Constipation	0.014394	2.52 × 10^−05^	TRUE	3.78 × 10^−42^
*g_Eubacterium rectale group*	Constipation	0.011963	2.56 × 10^−05^	TRUE	5.82 × 10^−35^
*g_Haemophilus*	Constipation	0.014611	1.82 × 10^−05^	TRUE	2.51 × 10^−43^
*g_Holdemanella*	Constipation	0.016530	2.51 × 10^−05^	TRUE	1.71 × 10^−48^
*g_Ruminococcus gnavus group*	Constipation	0.016941	2.64 × 10^−05^	TRUE	1.28 × 10^−49^
*unknown genus*	Constipation	0.016008	4.19 × 10^−05^	TRUE	7.26 × 10^−46^
*o_Methanobacteriales*	Constipation	0.013635	2.04 × 10^−05^	TRUE	2.99 × 10^−40^

*r*^2^, variance explanation rate.

SNP = single nucleotide polymorphism.

### 3.3. Sensitivity analysis

Heterogeneity of significant results was assessed using Cochran *Q* test and *I*² statistics (Table 4). The results indicate that the majority of the MR estimates for constipation did not exhibit significant heterogeneity (Cochran *Q P*-value >.05) and had low levels of heterogeneity (*I*² <50%). The result for the unknown genus was excluded from further analysis due to insufficient information.

**Table 4:** Heterogeneity testing in Mendelian randomization analysis of gut microbiota and constipation.

Exposure	Outcome	*Q*	*Q*_df	*Q*_*P*-val	*I*^2^ (%)
*c_Betaproteobacteria*	Constipation	2.967119	9	.965584	0
*c_Methanobacteria*	Constipation	5.272467	8	.728097	0
*f_Methanobacteriaceae*	Constipation	5.272467	8	.728097	0
*g_Coprococcus2*	Constipation	16.868972	7	.018261	41.50
*g_Eubacterium brachy group*	Constipation	6.130461	9	.726794	0
*g_Eubacterium rectale group*	Constipation	5.413628	6	.393591	0
*g_Haemophilus*	Constipation	4.123432	8	.845819	0
*g_Holdemanella*	Constipation	7.314864	10	.695423	0
*g_Ruminococcus gnavus group*	Constipation	8.117131	10	.617397	0
*unknown genus*	Constipation	10.447662	10	.402134	95.72
*o_Methanobacteriales*	Constipation	5.272467	8	.728097	0

*I*^2^, the statistics reflect the heterogeneity of the instrumental variables as a proportion of the total variation.

*Q* = Cochran *Q* test statistics; *Q*_df = Cochran *Q* test variance; *Q*_*P*-val = *P*-value of *Q* test.

To evaluate the horizontal pleiotropy of the instruments, MR-Egger regression analysis was performed for each indicator, with hypothesis testing results showing *P*-values for the intercepts >.05 and intercepts close to zero. This suggests that the causal inferences in this study are not influenced by horizontal pleiotropy (Table 5).

**Table 5:** Level pleiotropy test for Mendelian randomization analysis of intestinal microbiota for constipation.

Exposure	Outcome	Egger_intercept	Standard error	*P*-val
*c_Betaproteobacteria*	Constipation	−0.011701	0.010629	.302963
*c_Methanobacteria*	Constipation	−0.019881	0.016126	.257419
*f_Methanobacteriaceae*	Constipation	−0.019881	0.016126	.257419
*g_Coprococcus2*	Constipation	0.007339	0.044422	.874208
*g_Eubacterium brachy group*	Constipation	0.008259	0.012533	.528411
*g_Eubacterium rectale group*	Constipation	−0.017636	0.012681	.213694
*g_Haemophilus*	Constipation	−0.003315	0.008736	.715618
*g_Holdemanella*	Constipation	0.007266	0.008982	.439414
*g_Ruminococcus gnavus group*	Constipation	−0.020987	0.015204	.200789
*unknown genus*	Constipation	0.018354	0.014615	.240788
*o_Methanobacteriales*	Constipation	−0.019881	0.016126	.257419

By identifying and excluding outliers, we confirmed that none of the SNPs used in this study were outliers.

Sensitivity analysis was conducted using the leave-one-out method, where each line represents the effect size and 95% confidence interval range after excluding the corresponding SNP. The leave-one-out plot demonstrates that there is potentially influential SNPs driving the causal link between gut microbiota and constipation, and the current findings need to be interpreted carefully with caution.

### 3.4. MVMR analysis

Initially, univariate MR analyses were conducted to examine the significant causal effects of gut microbiota on constipation, alongside PD indicators. Significant causal relationships were identified using the IVW model and are presented in Table 6. Additionally, all Steiger directionality test results were TRUE, with *P*-values <.05, indicating no evidence of reverse causation (Table 7).

**Table 6:** MR causal effect estimates of gut microbiota and Parkinson disease (using the IVW random effects model).

Exposure	Outcome	Number of SNPs	β	Standard error	*P*-value	OR 95% CI
*c_Betaproteobacteria*	Parkinson disease	8	1.660022	0.619535	.036562	5.259426 (1.561634–17.713224)
*c_Methanobacteria*	Parkinson disease, strict definition	11	−0.547103	0.225508	.015262	0.578624 (0.371912–0.900227)
*f_Methanobacteriaceae*	Parkinson disease, strict definition	10	−1.325074	0.433032	.015583	0.265783 (0.113743–0.621058)
*g_Coprococcus2*	Parkinson disease, strict definition	10	−1.325074	0.433032	.015583	0.265783 (0.113743–0.621058)
*g_Eubacterium brachy group*	Parkinson disease, strict definition	10	−1.325074	0.433032	.015583	0.265783 (0.113743–0.621058)

β, Mendelian randomization analysis effect coefficient.

CI = confidence interval, OR = odds ratio, SNP = single nucleotide polymorphism.

**Table 7:** Steiger directionality test for Mendelian randomization analysis of the effect of gut microbiota on Parkinson disease.

Exposure	Outcome	SNP *r*^2^ exposure	SNP *r*^2^ outcome	Correct causal direction	Steiger *P*-value
*c_Betaproteobacteria*	Parkinson disease	0.017874	2.54 × 10^−05^	TRUE	7.30 × 10^−51^
*c_Methanobacteria*	Parkinson disease, strict definition	0.016749	0.000178	TRUE	1.41 × 10^−39^
*f_Methanobacteriaceae*	Parkinson disease, strict definition	0.015062	0.000147	TRUE	4.84 × 10^−36^
*g_Coprococcus2*	Parkinson disease, strict definition	0.015062	0.000147	TRUE	4.84 × 10^−36^
*g_Eubacterium brachy group*	Parkinson disease, strict definition	0.015062	0.000147	TRUE	4.84 × 10^−36^

*r*^2^, variance explanation rate.

SNP = single nucleotide polymorphism.

**Figure 3.**
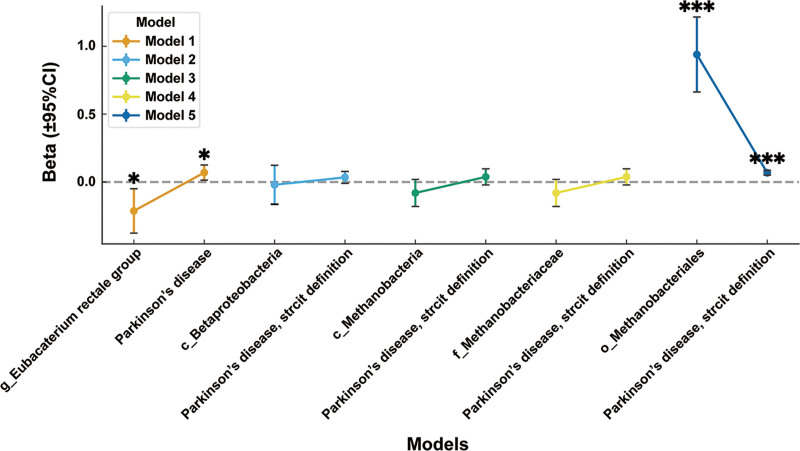
MVMR estimates for the effects of gut microbiota and PD on constipation across 5 models. Causal effect estimates (β, ±95% CI) were obtained using the IVW method. Five MVMR models (Model 1–Model 5) were constructed with different covariate adjustments. Each colored line represents 1 model, and points indicate causal effect estimates for the exposure–outcome pairs. Gut microbiota (e.g., *g_Eubacterium rectale group, c_Betaproteobacteria, c_Methanobacteria, f_Methanobacteriaceae, o_Methanobacteriales*) and PD were included as exposures, while constipation was used as the outcome. Asterisks denote statistical significance (*P <.05, **P <.01, ***P <.001). CI = confidence interval, IVW = inverse-variance-weighted, MVMR = multivariable Mendelian randomization, PD = Parkinson disease.

Subsequently, MVMR analyses were performed using these significant results as exposures to evaluate their association with constipation (Fig. 3). A total of 8 meaningful multivariable MR models were identified (Table 8). The results demonstrated a significant association between PD indicators and constipation in Model 1 and Model 5 (*P* <.05), while no significant associations were observed in the remaining models (*P* >.05).

**Table 8:** Multivariable Mendelian randomization analysis of the effects of gut microbiota and Parkinson disease on the onset of constipation.

Model	Exposure	Outcome	β	Standard error	*P*-value
Model1	*g_Eubacterium rectale group*	Constipation	−0.213158	0.083367	.010562
	Parkinson disease	Constipation	0.069284	0.028456	.014905
Model2	*c_Betaproteobacteria*	Constipation	−0.020570	0.073197	.778695
	Parkinson disease, strict definition	Constipation	0.034236	0.022233	.123590
Model3	*c_Methanobacteria*	Constipation	−0.080634	0.050886	.113057
	Parkinson disease, strict definition	Constipation	0.037691	0.030414	.215240
Model4	*f_Methanobacteriaceae*	Constipation	−0.080634	0.050886	.113059
	Parkinson disease, strict definition	Constipation	0.037691	0.030414	.215240
Model5	*o_Methanobacteriales*	Constipation	0.939216	0.140827	2.57 × 10^−11^
	Parkinson disease, strict definition	Constipation	0.068156	0.008863	1.47 × 10^−14^

β, the effect coefficients in MVMR analysis.

MVMR = multivariate Mendelian randomization, SNP = single nucleotide polymorphism.

### 3.5. Mediation analysis

In the mediation effect assessment of the MVMR analysis, models with significant causal relationships between the mediator and the outcome were evaluated for mediation effects. According to the MVMR analysis results, significant effects of PD indicators on constipation were observed in Model 1 and Model 5 (*P* <.05). Therefore, the discussion focuses primarily on the potential mediation effects in these 2 models.

**Figure 4.**
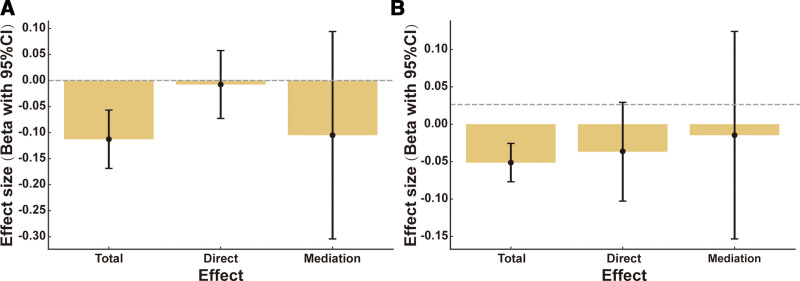
Mediation analysis of the effects of gut microbiota and PD on constipation. Estimated total, direct, and mediation effects (β, with 95% CI) are shown for 2 representative models. (A) Model 1: the effect of *g_Eubacterium rectale group* on constipation, with PD as the mediator. (B) Model 5: the effect of *o_Methanobacteriales* on constipation, with PD as the mediator. Effect estimates were obtained using the IVW method in MVMR. Bars represent effect sizes, and error bars indicate 95% confidence intervals. CI = confidence interval, IVW = inverse-variance-weighted, MVMR = multivariable Mendelian randomization, PD = Parkinson disease.

In both Model 1 and Model 5, we found that the direct effect estimated in MVMR does not equal the total effect calculated in 2-sample MR, suggesting that both models may constitute partial mediation effects (Fig. 4). Based on the direct effect estimates from the univariate MR analysis of gut microbiota on PD indicators and the direct effect estimates from the multivariable MR analysis of PD indicators on constipation development, the indirect effects of gut microbiota on constipation via PD indicators were calculated (Table 9). The results showed that the indirect effect size of gut microbiota on constipation in Model 1 and Model 5 shares the same sign as their direct effect size. The ratio of the indirect effect size to the direct effect size of gut microbiota on constipation is 193.17% in Model 1 and 128.44% in Model 5.

**Table 9:** Evaluation of Mendelian randomization mediated effect of gut microbiota mediated Parkinson Disease indicators on the onset of Constipation (partial mediation).

Model	Exposure	Mediator	Outcome	Direct effect E-M (95% CI)	Direct effect M-O (95% CI)	Mediation effect (95% CI)	Direct effect E-O (95% CI)	Total effect E-O (95% CI)	Mediation effect/total effect E-O*100 %
1	*g_Eubacterium rectale group*	Parkinson disease	Constipation	1.66 (1.04–2.28)	0.01 (0.00–0.02)	−0.11 (−0.30 to 0.09)	−0.01 (−0.07 to 0.06)	−0.11 (−0.17 to −0.06)	193.17%
5	*o_Methanobacteriales*	Parkinson disease, strict definition	Constipation	−1.33 (−1.76 to −0.89)	0.01 (−0.04 to 0.06)	−0.01 (−0.15 to 0.12)	−0.04 (−0.10 to 0.03)	−0.05 (−0.08 to −0.03)	128.44%

CI = confidence interval.

## 4. Discussion

Constipation is a complex disorder with intricate mechanisms that remain not fully understood. A growing body of evidence suggests that the gut microbiota plays a pivotal role in the pathophysiology of constipation. It is known that autonomic dysfunction of the digestive tract, slow gastrointestinal motility, and gut microbiota dysbiosis caused by PD are associated with the onset of constipation.^[26–28]^ This study aims to investigate the role of gut microbiota in PD-related constipation symptoms, with a particular focus on the involvement of *g_Eubacterium rectale group* and *o_Methanobacteria* in this process.

A substantial body of research has revealed a significant association between PD symptoms and susceptibility to constipation, and gut microbiota dysbiosis has been consistently observed in PD patients, characterized by a lower abundance of *Eubacterium rectale*.^[29,30]^ However, the specific causal interactions among gut microbiota, PD, and constipation remain unclear. In this study, both univariable MR and MVMR analyses provided complementary insights, consistently implicating the *g_Eubacterium rectale group* and *o_Methanobacteria* in the pathogenesis of constipation in PD. Additionally, this study conducted a mediation analysis to evaluate the mediating role of gut microbiota composition in the relationship between PD and constipation.

The findings of this study indicate a significant causal relationship between changes in the composition of specific gut microbiota and the occurrence of constipation. Certain bacteria, such as *g_Eubacterium rectale group* and *g_Coprococcus2*, were shown to have a protective effect against the risk of developing constipation, while others, such as *o_Methanobacteria* and *g_Ruminococcus gnavus*, were associated with an increased risk. A growing body of evidence suggests that microbial metabolites, particularly short-chain fatty acids (SCFAs) and neurotransmitters, play critical roles in regulating gut function. SCFAs enhance intestinal motility, while serotonin (5-HT), produced by enterochromaffin cells and modulated by gut microbiota, is closely linked to the enteric nervous system and influences peristalsis.^[31,32]^ In addition to 5-HT, dopamine also plays a pivotal role in gut-brain communication. Approximately half of the body’s dopamine is synthesized in the gastrointestinal tract, largely by enterochromaffin cells, and its bioavailability is shaped by gut microbial activity. Moreover, gut microbiota such as *Lactobacillus* and *Bifidobacterium* have been implicated in modulating dopamine metabolism, while reduced dopamine levels may reciprocally alter microbial composition.^[33–35]^ In PD, central dopaminergic neuron loss may converge with impaired peripheral dopamine signaling, collectively exacerbating gastrointestinal dysmotility and constipation. Within the SCFAs, butyrate is of particular importance. *Eubacterium rectale* is a major butyrate-producing bacterium that plays a critical role in maintaining intestinal homeostasis. Butyrate promotes the contractility of intestinal smooth muscles, preserves the integrity of the gut barrier, and provides energy to colonic epithelial cells.^[36]^ Reduced abundance of *Eubacterium rectale* leads to decreased butyrate production, resulting in mucosal damage, chronic, low-grade inflammation, and impaired autonomic and muscular function of the gut, ultimately causing constipation.^[37]^ Additionally, the gut microbiota and its metabolites can influence constipation by modulating signaling pathways related to the “gut-brain axis.”^[17]^

In recent years, research has increasingly focused on the connection between gut microbiota, their metabolites, and PD. The pathological features of PD include abnormal deposition of α-Syn in both the central and enteric nervous systems and the loss of dopaminergic neurons in the substantia nigra (SN).^[38,39]^ Numerous studies have suggested that abnormal α-Syn aggregation in the gut of PD patients is associated with the gut microbiota and their metabolites.^[40,41]^ Notably, the reduced abundance of anti-inflammatory bacteria such as *Eubacterium rectale* may influence the development of PD through the “gut-brain axis.”^[42]^ A lower abundance of *Eubacterium rectale* in the gut can compromise the intestinal mucosal barrier, potentially increasing intestinal permeability. This may facilitate the transmission of gut-derived inflammation or toxins to the brain via the vagus nerve or bloodstream, triggering or exacerbating neuroinflammation, thereby further affecting gut immune regulation and the central nervous system.

Additionally, studies have shown that butyrate, produced by *Eubacterium rectale*, can regulate the immune system by activating G-protein-coupled receptors (such as GPR41 and GPR43) and inhibiting inflammatory responses in both the nervous system and gut, thus exerting a protective effect against the onset and progression of PD.^[43]^ Butyrate also has the potential to decrease blood-brain barrier (BBB) permeability, reduce microglial activation, and alleviate PD symptoms by influencing DNA methylation and regulating gene expression.^[44]^ Furthermore, butyrate helps maintain the health of enteric neurons, improves gut motility, and reduces intestinal inflammation, which may indirectly protect the central nervous system. However, studies have found that butyrate levels in the fecal samples of PD patients are significantly lower than those in healthy controls.^[29,45]^ This reduction may be related to changes in the composition and function of the gut microbiota in PD patients. Additionally, the decrease in butyrate levels has been linked to constipation symptoms, suggesting that butyrate produced by gut microbiota may influence the clinical manifestations of PD by modulating host epigenetic states and immune responses.^[46]^ This finding underscores the critical role of the gut microbiota in maintaining neurological health and suggests that restoring the balance of beneficial gut microbes could be a potential therapeutic strategy for PD.

*Methanobacteria* are a common group of anaerobic microorganisms in the human gut, primarily represented by *Methanobrevibacter smithii* and *Methanosphaera stadtmanae*. These microbes convert hydrogen (H_2_) and carbon dioxide (CO_2_) into methane (CH_4_) via methanogenesis pathways.^[47]^ In the gut ecosystem, *Methanobacteria* influence gas production, the composition of gut microbiota, and intestinal motility through their unique metabolic activities. Studies suggest that an increased abundance of *Methanobacteria* may be associated with a thicker intestinal mucus layer, potentially reducing its permeability and affecting the efficiency of stool passage through the intestines.^[48]^ A thicker mucus layer could lead to hardened stools and the development of constipation. Additionally, the proliferation of *Methanobacteria* consumes more H_2_ and CO_2_ to produce more CH_4_,^[36]^ thereby reducing gas expulsion and increasing pressure and bloating within the gut, which in turn inhibits intestinal peristalsis.^[48]^

Clinical studies have shown a strong association between CH_4_-positive breath tests and prolonged colon transit times, indicating that elevated CH_4_ levels in the gut may be linked to constipation.^[49]^ CH_4_ inhibits the contraction of longitudinal muscles in the proximal colon by activating voltage-dependent potassium channels and increasing IKV, thereby prolonging colonic transit time and contributing to constipation.^[50,51]^ Moreover, within the gut ecosystem, *Methanobacteria* coexist with other hydrogen-producing probiotic bacteria. However, their proliferation can disrupt this balance, reducing the production of SCFAs, which in turn affects the ability of enterochromaffin (EC) cells to produce serotonin (5-HT). This disruption can impair normal neurochemical signaling between the gut and brain.^[52]^ Furthermore, studies have indicated that altered 5-HT levels are associated with emotional and behavioral changes in PD patients.^[53,54]^

The results of the univariable MR analysis indicated a negative association between the increase in *Methanobacteria* and constipation symptoms, as well as a negative correlation with the risk of PD. However, the multivariable MR analysis revealed contrasting findings, showing that an increase in *Methanobacteria* is associated with a higher risk of PD and promotes constipation symptoms. This discrepancy suggests that *Methanobacteria*, as major inhibitors of butyrate production, may influence intestinal permeability. Numerous studies have demonstrated a positive correlation between CH_4_ levels and both the incidence and severity of gastrointestinal motility disorders, such as constipation.^[55–57]^ However, some studies also suggest that when *Methanobacteria* (such as *Methanobrevibacter smithii*) are present at low concentrations in the gut, their symbiotic competition can protect the intestinal barrier by reducing the production of harmful metabolites like H_2_S.^[47]^

Studies have demonstrated an association between gut microbiota and PD, with particular attention given to changes in *Methanobacteria*. PD is characterized by abnormal deposition of α-synuclein (α-Syn) in both the central nervous system and the enteric nervous system, as well as the loss of dopaminergic neurons in the SN.^[38,39]^ Numerous studies have shown that abnormal aggregation of α-Syn in the intestines of PD patients is associated with alterations in gut microbiota and their metabolites.^[40,41]^ However, the composition of gut microbiota in PD patients is generally found to be dysregulated,^[29]^ potentially creating an environment conducive to the development of neurodegenerative diseases like PD.^[58]^ Research indicates that in the context of gut dysbiosis in PD patients, the proliferation of *Methanobacteria* can inhibit the growth of beneficial bacteria, such as butyrate-producing bacteria, leading to a reduction in butyrate levels and exacerbation of PD symptoms. Under these pathological conditions, there may also be misfolding and aggregation of α-Syn, which can then retrogradely propagate along the vagus nerve to the central nervous system.^[14,59]^ Moreover, methane produced by *Methanobacteria* may alter gut smooth muscle activity and motility, affecting intestinal permeability and compromising the intestinal mucosal barrier. This increased permeability can allow gut-derived toxins, such as lipopolysaccharides (LPS), to enter the bloodstream, thereby increasing BBB permeability and influencing PD pathogenesis.^[13,60]^ LPS in the bloodstream may activate the immune system, particularly through innate immune receptors like TLR4, inducing the release of pro-inflammatory cytokines such as IL-6 and TNF-α, which can exacerbate neuroinflammation and trigger systemic inflammatory responses, leading to neuroinflammatory damage.^[61]^ Furthermore, the accumulation of excess methane in the gut due to *Methanobacteria* proliferation and the resultant pro-inflammatory state may be associated with neurodegenerative changes, potentially further influencing the progression of PD. Methane may also affect the activity of the enteric nervous system, alter the transport of small or large molecules (such as prion-like propagation of α-Syn) along the vagus nerve, or impact electrical signaling and neurotransmitter transmission, thereby affecting the central nervous system.^[39,62]^ Such changes could further exacerbate both motor and non-motor symptoms in PD patients. Therefore, understanding the role of *Methanobacteria* in the gut microbiome and its regulatory mechanisms may provide new insights and targets for the early intervention and treatment of PD.

Previous studies have highlighted the relationship between gut microbiota, PD, and susceptibility to constipation.^[63]^ Given the complex interplay within the gut-brain-microbiota axis, metabolites produced by gut microbiota, such as butyrate, gamma-aminobutyric acid (GABA), and serotonin (5-HT), may be linked to PD and intestinal epithelial inflammation.^[64]^ However, the precise relationships or causal links among these 3 factors remain unclear. Using MVMR analysis following univariable MR analysis with significant results for constipation as the exposure factor, our study found that patients exposed to the *o_Methanobacteria* and at risk for PD were more likely to develop constipation, whereas exposure to the *g_ Eubacterium rectale group* appeared to have a protective effect.

With the advent of the “gut-brain axis” theory, numerous studies have suggested that an imbalance in the gut microbiota may influence the progression of PD through the gut-brain axis.^[27,65]^ Fecal microbiota transplantation (FMT), an emerging therapeutic approach, has been shown to be effective in treating constipation. This therapy involves transplanting fecal microbiota from healthy donors into patients to restore the balance of gut microbiota and alleviate symptoms in PD patients, particularly constipation. Extensive animal studies have demonstrated that FMT significantly restores gut microbial communities, alleviates gut inflammation and barrier disruption, and improves gastrointestinal dysfunction and motor deficits in PD models, while also reducing systemic inflammation. FMT treatment has also been shown to reduce BBB damage, lower lipopolysaccharide (LPS) levels in the colon, serum, and SN, and inhibit neuroinflammation in the SN by reducing the expression of components of the TLR4/TNF-α signaling pathway in the gut and brain, thereby mitigating dopaminergic neuronal damage.^[66–68]^ Additionally, studies have indicated that constipation patients exhibit specific gut microbiota profiles, and modulating their gut microbial composition through FMT can alleviate both PD and constipation symptoms.^[69]^ These findings suggest that FMT effectively relieve symptoms of both constipation and PD by modulating interactions between the brain and gut microbiota. Our MVMR analysis further highlights the interplay between gut microbiota and PD in the pathogenesis of constipation. This aligns with emerging evidence that indicates a bidirectional communication between the central nervous system and gut microbiota via the “gut-brain axis,” which may influence both brain disorders and gastrointestinal function.

The significant causal relationships observed in Models 1 and 5 further support the critical role of PD in the pathogenesis of constipation. This finding is consistent with existing literature, suggesting that PD may drive changes in gut microbiota in certain patients. These findings not only confirm the mediating role of PD in the relationship between gut microbiota and constipation but also highlight the heterogeneity of this association. Amid uncertainties about the mechanisms underlying constipation, our results provide new insights into the complex interactions among PD, gut microbiota, and constipation. The mediating effect of PD on the relationship between gut microbial composition and constipation underscores the importance of considering neurological aspects in managing gastrointestinal disorders. The findings suggest that interventions targeting neurological conditions may help reduce the risk of constipation associated with an unfavorable gut microbiota composition.

There are, however, some limitations to this study that should be considered. First, the gut microbiota data used in this study were derived from the Dutch Microbiome Project, which may introduce potential analytical bias due to inherent heterogeneity in dietary habits, geographical environments, gut microbiota sampling, or sequencing methods. Consequently, the results of this study may have a certain margin of error. Second, constipation encompasses various subtypes, such as functional constipation, organic constipation, and drug-induced constipation. Previous studies have shown differences in gut microbiota among these subtypes, but this study did not explicitly classify them. Instead, all subtypes were analyzed as a whole, rather than individually. Future research should explore the relationships among PD, gut microbiota, and different constipation subtypes. Moreover, this study lacks detailed data on clinical characteristics, such as constipation symptom scores, dietary patterns, and previous gastrointestinal infection history. The absence of such data may limit the depth of analysis and interpretation of the study’s findings. Finally, this study lacks direct evidence to establish a causal association and mediating effect between constipation, PD, and gut dysbiosis. Future research is needed to validate the potential role of these microbes in the development of constipation and neurodegenerative diseases through the gut-brain axis.

## 5. Conclusion

This study not only methodologically validates the causal relationship between PD-mediated gut microbiota and the risk of constipation but also provides new insights for advancing constipation research. Future studies should focus on elucidating the dynamic interactions between gut microbiota, PD, and constipation, as well as investigating their molecular mechanisms to identify more precise clinical therapeutic targets. Notably, this study reveals that *Eubacterium rectale* and *Methanobacteria* may influence the gut-brain axis by acting on butyrate, suggesting that future research should delve deeper into the microecological study of gut microbiota to further understand the role of microbes in the progression of PD.

However, it is important to note that the pathogenesis of constipation is far from fully understood, and there are variations among different research findings. Current research faces several challenges, including sample heterogeneity, differences in study design, and the need to address the high interindividual variability of gut microbiota. Thus, this study provides a novel perspective on the complex mechanisms by which PD-mediated gut microbiota influence constipation. It not only confirms the causal relationship between PD and constipation at the data level but also adds new supporting evidence to the existing literature on the associations among gut microbiota, PD, and constipation, while emphasizing their diversity.

## Acknowledgments

We would like to acknowledge the hard and dedicated work of all the staff that implemented the intervention and evaluation components of the study.

## Author contributions

**Conceptualization:** Jiamu Zheng, Beibei Xu, Wenhuan Wang.

**Data curation:** Jiamu Zheng, Beibei Xu, Wenhuan Wang.

**Formal analysis:** Jiamu Zheng, Beibei Xu, Wenhuan Wang.

**Funding acquisition:** Lidan Gao.

**Investigation:** Jiamu Zheng, Wenhuan Wang.

**Methodology:** Jiamu Zheng, Beibei Xu, Wenhuan Wang.

**Project administration:** Jiamu Zheng, Beibei Xu, Wenhuan Wang.

**Resources:** Jiamu Zheng, Beibei Xu, Lidan Gao, Wenhuan Wang.

**Software:** Jiamu Zheng, Lidan Gao, Wenhuan Wang.

**Supervision:** Jiamu Zheng, Beibei Xu, Wenhuan Wang.

**Validation:** Jiamu Zheng, Beibei Xu, Wenhuan Wang.

**Visualization:** Jiamu Zheng, Beibei Xu, Wenhuan Wang.

**Writing – original draft:** Jiamu Zheng.

**Writing – review & editing:** Beibei Xu, Wenhuan Wang.
